# Rural-urban differentials of premature mortality burden in south-west China

**DOI:** 10.1186/1475-9276-5-13

**Published:** 2006-10-14

**Authors:** Le Cai, Virasakdi Chongsuvivatwong

**Affiliations:** 1 191 western Renmin road, Department of Health Information and Economics, Faculty of Public Health, Kunming Medical College, Kunming 650031, China; 2 Epidemiology Unit, Faculty of Medicine, Prince of Songkla University, Hat Yai, Songkhla 90112, Thailand

## Abstract

**Background:**

Yunnan province is located in south western China and is one of the poorest provinces of the country. This study examines the premature mortality burden from common causes of deaths among an urban region, suburban region and rural region of Kunming, the capital of Yunnan.

**Methods:**

Years of life lost (YLL) rate per 1,000 and mortality rate per 100,000 were calculated from medical death certificates in 2003 and broken down by cause of death, age and gender among urban, suburban and rural regions. YLL was calculated without age-weighting and discounting rate. Rates were age-adjusted to the combined population of three regions. However, 3% discounting rate and a standard age-weighting function were included in the sensitivity analysis.

**Results:**

Non-communicable diseases contributed the most YLL in all three regions. The rural region had about 50% higher premature mortality burden compared to the other two regions. YLL from infectious diseases and perinatal problems was still a major problem in the rural region. Among non-communicable diseases, YLL from stroke was the highest in the urban/suburban regions; COPD followed as the second and was the highest in the rural region. Mortality burden from injuries was however higher in the rural region than the other two regions, especially for men. Self-inflicted injuries were between 2–8 times more serious among women. The use of either mortality rate or YLL gives a similar conclusion regarding the order of priority. Reanalysis with age-weighting and 3% discounting rate gave similar results.

**Conclusion:**

Urban south western China has already engaged in epidemiological pattern of developed countries. The rural region is additionally burdened by diseases of poverty and injury on top of the non-communicable diseases.

## Background

The allocation of health care resources has become an important issue in many countries experiencing resource limitation, and the assessment of the burden of disease (BOD) is useful for sensible allocation of limited health-related resources [1]. China is a developing country where health resources are very scarce and inequality in health resource allocation between rural and urban regions is apparent [2,3]. Urban populations, which account for only 30% of whole population, use 80% of total health resources, whereas rural populations, which account for 70% of total populations, only use 20% of total health resources [4].

Yunnan province is located in the south-west of China, and is one of the poorest provinces in the country. In 2004 the per capita GDP of Yunnan province was US$680, which was 37.6% lower than that of the whole country [5]. Kunming prefecture, the capital, contains two urban, two suburban and eight rural regions, and in 2000 had a population of approximately 5.8 million.

There are three well established systems for death registry in Kunming prefecture, located in an urban region, a suburban region and a rural region respectively. The first two are the surveillance points of death registry of China, whereas the other is one of the surveillance points of the country birth registration. In order to maintain accuracy and completeness of birth and death registration, an independent resurvey on a sample of about 5000 households once every three years is conducted in each of these surveillance points. Compared to the surveillance data, the 2000 census in China actually had more underreported deaths, and was less complete due to the problem of recall bias. These well defined regions and established registration systems provide a good ground for comparison of mortality burden across the three types of regions along with economic transition.

Years of life lost (YLL) is one of the methods of estimating the duration of time lost due to premature death, and is the mortality component of disability adjusted life years (DALY). The conceptual and computational details of years of life lost have been presented elsewhere [6]. The YLL measure not only considers the number of deaths, but also takes into account the age at which death occurred. It is therefore a better tool for quantifying the burden of premature mortality compared to mortality rate per se.

The purpose of this study is to compare the premature mortality burden from each common cause among an urban region, suburban region and rural region of Kunming, broken down by age and gender. This assessment should provide more insight into the details of health inequity across the rural/urban gradient.

## Methods

### Study populations

Pan Long district, Guan Du district and Shi Lin county were selected as the study regions. Pan Long district is an urban region, with a population of 378,885 (195,590 males and 183,295 females); Guan Du district is a suburban region, with a population of 335,622 (178,099 males and 157,523 females), and Shi Lin county a rural region with a population of 179,746 (93,327 males and 86,419 females). All individuals residing and dying in these three regions in 2003 were included in the analysis.

### Data source

Causes of death were based on medical death certificate information, maintained by Pan Long Center for Diseases Control, Guan Du Center for Diseases Control and Shi Lin maternal and children hospital. All death reports were grouped by underlying cause of death as defined in the Global Burden of Disease (GBD) study [7] and coded using the International Classification of Diseases, 9th revision (ICD-9) coding system.

In order to avoid some misreporting of age at death, and misclassification of cause of death, all medical death certificates were verified on the underlying cause of death by a team of two independent physicians. Any discrepancies were reviewed to obtain a consensus. All deaths assigned to ill-defined conditions among the three study regions were redistributed to other more defined causes according to the age and gender distribution of specific conditions, following the conceptual approach in the GBD study [8].

Overall mortality in Kunming was divided into three broad groups of causes: Group I, communicable, maternal, perinatal and nutritional deficiencies; Group II, non-communicable; and Group III, all injuries. These were then further subdivided into several more specific causes [8]. Age was divided into five groups: 0–4, 5–14, 15–44, 45–59 and 60 years and over. Overall age-specific mortality for each sex was plotted for visual comparison.

### Calculation of Years of Life lost (YLL) and mortality rate

In global burden of disease studies, YLL incorporates an age-weighting factor so that life lived in young adults is given a higher social value than that in childhood or older ages in most societies [9,10]. However, the use of age-weighting has been criticized by some researchers [11]. Traditionally, Chinese culture values living years in the elderly and children, so age-weighting was not used in this study. This study calculated YLL without discounting rate, because deaths occurred in each calendar year and the study has a cross-sectional nature. YLL was also calculated with a 3% discounting rate and a standard age-weighting function. The results of the final ranking were the same so they were omitted from this report and only the YLL calculated without discounting and age-weighting presented. Nevertheless, 3% discounting rate and a standard age-weighting function were included in the sensitivity analysis.

The simplified formula of YLL without discounting and age weighting is:

YLL(0,0)=∑dxex
 MathType@MTEF@5@5@+=feaafiart1ev1aaatCvAUfKttLearuWrP9MDH5MBPbIqV92AaeXatLxBI9gBaebbnrfifHhDYfgasaacH8akY=wiFfYdH8Gipec8Eeeu0xXdbba9frFj0=OqFfea0dXdd9vqai=hGuQ8kuc9pgc9s8qqaq=dirpe0xb9q8qiLsFr0=vr0=vr0dc8meaabaqaciaacaGaaeqabaqabeGadaaakeaacqWGzbqwcqWGmbatcqWGmbatcqGGOaakcqaIWaamcqGGSaalcqaIWaamcqGGPaqkcqGH9aqpdaaeabqaaiabdsgaKnaaBaaaleaacqWG4baEaeqaaOGaemyzau2aaSbaaSqaaiabdIha4bqabaaabeqab0GaeyyeIuoaaaa@3D9E@

where *d*_*x *_is the number of deaths at age x, and *e*_*x *_is the life expectancy at that age.

Life expectancy was calculated using the life tables provided in the GBD study- the model life-table west level 26 [12], which is based on the life expectancy at birth of 82.5 years for females. West level 25 for females is used as the standard for males (in the absence of a Coale and Demeny life table with life expectancy of 80 for males), to ensure comparability with other studies.

Mortality rates were also calculated to compare with the use of YLL as an indicator of mortality burden.

Abridged life tables was used to calculate the average life expectancy at birth of three regions. Ninety-five percent confidence interval of the life expectancy was also computed using the Chiang methodology – Chiang (II) [13].

### Calculations for adjusted rate

The direct standardization method was used to calculate both the age-adjusted YLL rate and the age-sex-adjusted mortality rate. The total population of all 3 study regions was used as the standard population. For comparison of age-specific mortality and YLL rates, the sex distribution was adjusted using the same standard population.

## Results

The average life expectancy at birth was 77.1 years (95% CI: 76.5–77.8) for the urban region, 74.3 years (95% CI: 73.5–75.1) for the suburban region and 70.8 years (95% CI: 70.0–71.5) for the rural region.

There were 2,032 deaths registered in the urban region (56.1% males, 43.9% females), 1,927 deaths in the suburban region (53.1% males, 46.9% females) and 1,065 deaths (53.4% males, 46.6% females) in the rural region of Kunming for 2003. Thirty-two deaths in all three regions combined, which were coded as ill-defined conditions, were redistributed proportionally to other more defined causes. All of them were aged over 70 years.

**Figure 1:**
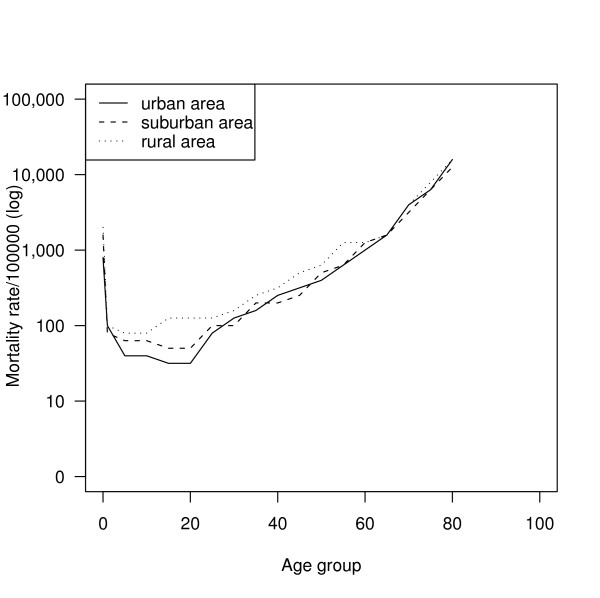
Comparison of male age-specific mortality in three regions.

**Figure 2:**
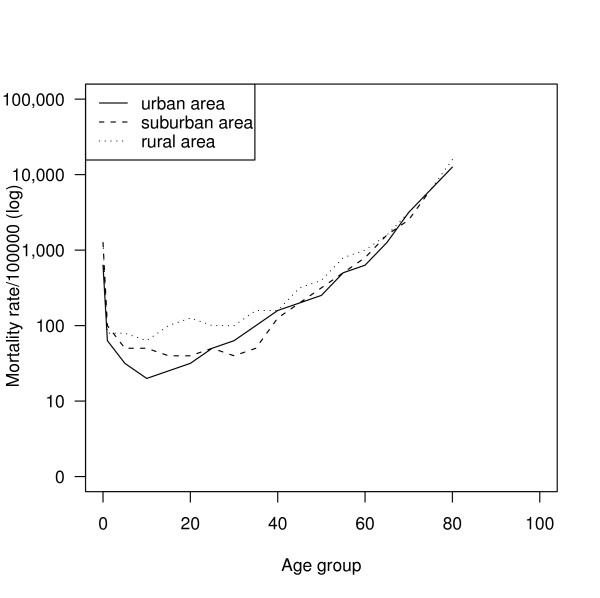
Comparison of female age-specific mortality in three regions.

The overall age-specific mortality rates by region are illustrated in Figure 1 (males) and Figure 2 (females). Mortality rates were higher in the rural region throughout the whole age range in both sexes. The urban population had the lowest mortality rate among the three regions in childhood and early adulthood. The rates were similar for all three regions after 60 years of age for both sexes.

Table 1 shows a comparison of YLL by broad cause groups among the three regions. Group I conditions were responsible for the highest mortality burden in the rural region compared to the other two regions, as were group III conditions. Group II conditions accounted for over 50% of the total YLL in each of the three regions, indicating that non-communicable diseases were the major premature mortality burden in the study regions as a whole.

**Table 1:** Mortality burden by cause among urban, suburban and rural region of Kunming, 2003

Causes of Death *	Urban region			Suburban region			Rural region		
			
	YLL	Age-adjusted YLL/1,000 population	%	YLL	Age-adjusted YLL/1,000 population	%	YLL	Age-adjusted YLL/1,000 population	%
		
Group I	1859	5.1	4.7	1791	5.8	4.6	4337	23.8	15.8
Group II	32854	85.1	82.3	30423	92.6	78.8	14880	106.7	54.2
Group III	5184	13.9	13.0	6418	20.5	16.6	8221	47.2	30.0
Total	39897	104.2	100.0	38631	122.9	100.0	27438	158.8	100.0

* Group I: communicable, maternal, perinatal and nutritional deficiencies

Group II: non-communicable

Group III: injuries

Table 2 presents age breakdown of sex-adjusted YLL/1,000 population by broad cause groups among the three regions. Group I accounted for the highest YLL/1,000 population in the 0–4 age group. Non-communicable diseases were responsible for the highest YLL/1,000 population in the 60+ age group among the three regions. The premature mortality from injuries was the highest in young adults aged 15–44 years in all three regions. The rural region had the highest premature mortality burden in all three death cause groups when the rate per thousand population was used.

**Table 2:** Sex-adjusted YLL/1,000 population by age, cause and region of Kunming, 2003

Age group	Group I **			Group II **			Group III **		
			
	Urban	Suburban	Rural	Urban	Suburban	Rural	Urban	Suburban	Rural
0–4	58.5	111.7	180.8	23.5	123.4	46.1	14.9	18.0	38.9
5–14	0.0	4.9	7.7	9.3	17.8	20.1	6.3	23.4	39.8
15–44	3.1	0.6	7.0	27.2	23.7	25.7	25.6	58.6	57.3
45–49	2.7	2.8	8.2	148.8	125.3	111.2	11.8	16.8	35.0
60+	3.3	4.2	8.4	585.3	511.0	540.4	14.4	18.5	26.1
All ages *	5.1	5.8	23.8	85.1	92.6	106.7	13.9	20.5	47.2

* age-adjusted

** Group I: communicable, maternal, perinatal and nutritional deficiencies

Group II: non-communicable

Group III: injuries

Table 3 compares YLL/1,000 population by sex and age among the three regions of Kunming. People aged 60 years and over were responsible for the highest years of life lost compared to the other age groups in each of the three regions. Children aged less than 5 years also had relatively high premature mortality burden in all three regions. In the age group of 60 years and over, the rural region had the lowest value of years of life lost, whereas in the 0–4 years age group, the urban region was responsible for the lowest premature mortality burden.

**Table 3:** YLL/1,000 population by age, gender and region of Kunming, 2003

Age group	Urban			Suburban			Rural		
			
	Male	Female	All*	Male	Female	All *	Male	Female	All*
0–4	143.4	76.5	107.9	220.6	342.0	258.1	298.1	191.6	265.8
5–14	22.8	10.0	15.6	44.8	47.4	46.1	69.6	37.5	67.7
15–44	59.7	34.4	46.0	50.4	30.5	42.8	78.6	58.3	70.1
45–59	109.2	82.8	101.7	107.2	87.8	104.9	129.2	96.9	122.0
60+	631.7	617.8	623.0	623.9	697.7	662.6	536.5	553.6	554.9
All ages **	128.2	82.7	104.2	136.5	108.0	122.9	185.9	129.3	158.8

* sex adjusted

** age adjusted

Table 4 presents expanded information of group of causes of death in terms of adjusted mortality rate/100,000 and YLL/1,000 by region. Although the figures of these two indicators are on a different scale, the ranking within the region are similar. However, there are some differences in ranking with regions. Birth trauma and asphyxia and low birth weight rank low by both indicators in urban and suburban regions, but rank relatively higher by both indicators in the rural region. For group I diseases, birth trauma and asphyxia and low birth weight were important leading causes of years of life lost in the rural region, and had a serious impact on premature health. For group II diseases, stroke was the top leading cause of years of life lost in both the urban and suburban regions, whereas it ranked third in the rural region. COPD was also a leading cause of premature death among the three regions, being the second ranked cause of years of life lost in the urban and suburban region, and the top cause of years of life lost in the rural region. Diabetes was a common problem in the urban region, while ischemic heart disease was predominant in the suburban region. Lung and liver cancer were major causes of premature death in the urban and suburban regions, but were less common in the rural region. For group III diseases, road traffic accidents were among the top 10 leading causes of premature death among all three regions, particularly the rural region, where it ranked fourth in both mortality and YLL. Self-inflicted injury was a major premature mortality concern in the rural region.

**Table 4:** Age-adjusted mortality and YLL by causes among three regions of Kunming, 2003

Cause group	Cause	Urban region		Suburban region		Rural region	
				
		Mortality^1 ^(rank)	YLL^2 ^(rank)	Mortality^1 ^(rank)	YLL^2 ^(rank)	Mortality^1 ^(rank)	YLL^2 ^(rank)
Group I							
	Birth trauma and asphyxia	0.8 (52)	0.7 (34)	3.3 (35)	2.0 (26)	16.9 (9)	6.8 (5)
	Low birth weight	0.6 (54)	0.4 (41)	2.0 (40)	1.2 (32)	9.4 (11)	4.6 (8)
Group II							
	Stroke	92.8 (1)	14.7 (1)	132.7 (1)	19.4 (1)	78.6 (2)	15.0 (3)
	COPD*	51.2 (2)	6.3 (2)	129.5 (2)	17.3 (2)	184.4 (1)	27.5 (1)
	Diabetes mellitus	34.3 (3)	5.7 (3)	11.6 (11)	2.2 (11)	4.1 (28)	1.3 (28)
	Ischemic heart disease	21.0 (6)	3.0 (10)	53.3 (3)	9.3 (3)	19.3 (8)	4.2 (10)
	Lung cancer	30.3 (4)	5.6 (4)	23.6 (4)	5.2 (5)	16.7 (10)	4.3 (9)
	Liver cancer	14.4 (8)	3.3 (8)	14.3 (6)	4.1 (7)	7.3 (17)	2.3 (18)
Group III							
	Road traffic accidents	10.4 (9)	4.2 (6)	11.7 (10)	4.6 (6)	32.9 (4)	13.4 (4)
	Self-inflicted injuries	5.2 (16)	2.2 (14)	13.6 (7)	5.3 (4)	41.9 (3)	19.1 (2)

All causes		536.3	104.2	558.5	122.9	674.5	158.8

* COPD = Chronic obstructive pulmonary disease

1 Age-adjusted/100,000 population

2 Age-adjusted/1,000 population

Table 5 and 6 are a breakdown of Table 4 into male and female. For group II conditions, stroke, COPD, diabetes, lung cancer and liver cancer in both sexes had similar rankings as listed in Table 4. Geographic discrepancy of group I conditions was also not different between males and females. However, in the urban region, females accounted for higher years of life lost due to diabetes than males, while in the rural region ischemic heart disease was a more common cause of premature death among females than males. In the suburban region, females had higher years of life lost due to self-inflicted injuries than males. Road traffic accidents had a substantial impact on the health of males in each of the three regions, and resulted in a 2 to 3 times higher burden of years of life lost among males compared to females. For all causes, the number of years of life lost was higher in males than females in all three regions.

**Table 5:** Age-adjusted mortality and YLL by cause among three regions of Kunming, 2003 (males)

Cause group	Cause	Urban region		Suburban region		Rural region	
				
		Mortality^1 ^(rank)	YLL^2 ^(rank)	Mortality^1 ^(rank)	YLL^2 ^(rank)	Mortality^1 ^(rank)	YLL^2 ^(rank)
Group I							
	Birth trauma and asphyxia	1.1 (51)	0.9 (29)	5.1 (20)	2.3 (17)	15.9 (9)	8.9 (6)
	Low birth weight	0.5 (52)	0.4 (40)	2.6 (28)	1.4 (30)	10.7 (10)	6.3 (8)
Group II							
	Stroke	95.5 (1)	17.1 (1)	129.9 (1)	21.4 (1)	77.8 (2)	16.2 (4)
	COPD*	62.1 (2)	8.4 (3)	124.0 (2)	18.8 (2)	179.4 (1)	31.4 (1)
	Diabetes mellitus	27.1 (4)	4.8 (6)	9.3 (11)	2.1 (15)	6.6 (26)	2.0 (26)
	Ischemic heart disease	23.7 (5)	3.8 (10)	43.2 (3)	7.5 (3)	10.9 (17)	2.6 (20)
	Lung cancer	40.9 (3)	7.7 (4)	31.9 (4)	7.3 (4)	23.8 (6)	5.7 (9)
	Liver cancer	18.6 (7)	4.4 (8)	17.6 (5)	5.1 (6)	11.2 (15)	3.4 (15)
Group III							
	Road traffic accidents	14.1 (8)	6.1 (5)	15.1 (6)	6.4 (5)	41.1 (3)	18.3 (2)
	Self-inflicted injuries	5.0 (19)	2.2 (19)	9.4 (10)	3.3 (9)	32.9 (4)	17.9 (3)

All causes		556.5	128.2	564.6	136.5	712.4	185.9

* COPD = Chronic obstructive pulmonary disease

1 Age-adjusted/100,000 population

2 Age-adjusted/1,000 population

**Table 6:** Age-adjusted mortality and YLL by cause among three regions of Kunming, 2003 (females)

Cause group	Cause	Urban region		Suburban region		Rural region	
				
		Mortality^1 ^(rank)	YLL^2 ^(rank)	Mortality^1 ^(rank)	YLL^2 ^(rank)	Mortality^1 ^(rank)	YLL^2 ^(rank)
Group I							
	Birth trauma and asphyxia	0.6 (49)	0.5 (38)	1.4 (38)	1.2 (30)	9.0 (10)	5.6 (6)
	Low birth weight	0.5 (51)	0.4 (39)	1.3 (37)	1.0 (31)	6.5 (12)	3.4 (10)
Group II							
	Stroke	90.5 (1)	12.2 (1)	135.9 (1)	17.2 (1)	79.8 (2)	13.7 (3)
	COPD*	38.3 (3)	4.1 (3)	134.9 (2)	15.7 (2)	189.1 (1)	23.2 (1)
	Diabetes mellitus	42.0 (2)	7.1 (2)	14.0 (8)	2.3 (11)	1.6 (35)	0.5 (38)
	Ischemic heart disease	13.1 (10)	2.1 (12)	64.8 (3)	10.4 (3)	27.7 (4)	6.0 (5)
	Lung cancer	18.5 (4)	3.3 (5)	13.7 (9)	2.7 (8)	11.7 (9)	2.7 (11)
	Liver cancer	9.7 (11)	2.4 (8)	11.5 (10)	3.1 (7)	3.0 (24)	1.0 (26)
Group III							
	Road traffic accidents	6.5 (13)	2.2 (11)	7.9 (12)	2.6 (10)	24.2 (5)	8.1 (4)
	Self-inflicted injuries	5.3 (14)	2.1 (13)	18.5 (4)	7.5 (4)	43.2 (3)	20.9 (2)

All causes		492.4	82.7	549.6	108.0	636.5	129.3

* COPD = Chronic obstructive pulmonary disease

1 Age-adjusted/100,000 population

2 Age-adjusted/1,000 population

## Discussion

These findings indicate that the epidemiologic transition is well under way in the study communities. For all three regions, the leading causes of premature death are non-communicable diseases. There are great health disparities across the rural/urban gradient with the rural region having about a 50% higher premature mortality burden compared to the other two regions. Using either mortality rate or YLL gives similar conclusion in order of priority. The rural region is still suffering from infectious diseases and prenatal problems, with five times the burden rates of the other two regions. Among the top non-communicable diseases, stroke is the most common mortality burden in the urban and suburban regions. COPD follows as the second highest burden and is the top burden in the rural region. Ischemic heart disease, although having a lower rate, still poses an important mortality burden in the suburban region. Mortality burden from injuries is however higher in the rural region than in the other two regions. For men living in rural regions, death from road traffic accidents is a major concern. However, self-inflicted injuries are two to eight times higher among women in this setting.

In the rural region, chronic diseases have not displaced but added to the mortality burden from infectious and perinatal problems, and this double burden is a major challenge for health systems in this setting. These results are consistent with other studies in China [14,15], and in other parts of the developing world, such as Pakistan [16]. Our results differed from a study in sub-Saharan Africa [17], where childhood and infectious diseases are still the major mortality burden. In urban Kunming, ischemic heart disease, stroke, diabetes, COPD and lung cancer are among the top 10 leading causes of YLL. The situation is similar to that in developed countries such as USA and Switzerland [18,19]. This may be partly explained by the higher socio-economic status, more hygienic life style and better access to health care. All of these suppress infectious diseases and prenatal problems but can lead to a higher life expectancy and therefore more chronic diseases. On the other hand, comparison among studies has to take into account the difference in discounting rate and age-weighting scheme. Our study calculated YLL without discounting rate and age-weighting, whereas those from developed countries such as US and Switzerland have used 3% discounting rate and age-weighting.

In the rural region, birth trauma and asphyxia and low birth weight are listed in the top 10 causes of YLL. This may be partly explained by the effect of the age distribution. In this study, the 0–1 age group occupies 1.7% of the whole population in the rural region, compared to 0.8% of the urban and 0.7% of the suburban population. The discrepancy in the proportions is due to a higher birth rate and lower life expectancy in the rural region [20]. This two-fold difference in percentage of the infant population however does not explain the four to five time differences in mortality burden of this age group across the rural-urban gradient. China has achieved fairly steady economic growth over the past 20 years. However, the greater disparities in economic development among different regions have led to health disparities among the urban and rural regions, especially for maternal and infant mortality [21,22].

The leading mortality burden for non-communicable diseases in our study regions include stroke, ischemic heart disease, diabetes, COPD, cancer and injuries. Stroke was a much higher mortality burden compared to ischemic heart disease. The dominance of stroke over ischemic heart disease has been repeatedly found in most parts of mainland China [23,24]. However, the situation is different from that among ethnic Chinese in Hong Kong and Singapore [25], and Taiwan [26], where ethnic Chinese die more from ischemic heart disease than from stroke. Heart disease ranks third among the leading causes of mortality in Taiwan, and is a more common cause of death than stroke. These differences are less likely to be explained by genetic backgrounds than environmental or behavioural factors.

Our findings show that COPD is a major cause of death particularly in the non-urban region. Results of the 1996 National Prevalence Survey in China indicated that more than 60% of men in China currently smoked, whereas this figure was less than 3% among women [27]. Tobacco is the biggest industry in Yunnan, accounting for 30% of China's tobacco production. In 2002 it was responsible for 17.6% of the national total output [28]. More than 2.3 million farmers in the province are engaged in tobacco cultivation. Our results indicate that COPD is a substantial cause of premature death for both genders among all three regions. This is partly explained by the high prevalence of smoking and the fact that COPD accounts for 35% of all smoking related deaths [29]. However, the females in our study had a slightly higher premature mortality burden due to COPD than males in the suburban and rural regions, pointing to the existence of risk factors other than smoking on COPD in women. In 2002 the World Health Organization estimated that 80% of Chinese households use biomass fuel, and coal used in 31% of households [30]. Solid-fuel use has been shown to be an important risk factor for COPD [31]. From household work, women are usually more exposed to this risk factor, which may contribute to the female premature mortality burden. The nature of this additional etiologic factor needs to be further explored.

This study demonstrates a higher premature mortality burden due to injuries in the rural region than in the urban region, especially fatal road traffic accidents among males. This could not be explained by the difference in rate of transportations, which is usually higher in urban regions. Several studies have indicated that the largest proportions of road traffic victims in developing countries are pedestrians, passengers, and cyclists as opposed to drivers, in whom most of the deaths and disabilities in the developed world occur [32,33]. Sharing of the road by high speed vehicles and walking villagers in addition to other factors of road traffic accidents may be an explanation for the rural predominance [34].

Self-inflicted injuries are a serious premature mortality burden, especially among females in both the suburban and rural region. In contrast, the rate of suicide is almost the same for men and women in the urban region. The situation is different from those in Micronesia, Hong Kong China and Singapore, where women die less often by suicide than men [35]. High suicide rates are due to multiple factors such as religious beliefs, social isolation, family problems, stressful life events, chronic painful diseases, mental disorders and substance abuse [36]. Psychiatric services have been shown to reduce the rate [37]. Further studies to identify the causes of these injuries and to improve the mental services are needed in our study region.

There are many methodological lessons from this study. Firstly, a good mortality registration system can be accomplished in a developing country such as China. Secondly, mortality rate gives similar results to the standard YLL computation for Group II and group III conditions in terms of priority ranking once the country is overwhelmed by a group of diseases such as diabetes, ischemic heart disease and stroke, which affect a relatively homogenous age group. However, in the longer term and over a broader area, YLL would still be more appropriate when the patterns of disease are less homogeneous. Thirdly, this kind of study can be useful in uncovering less known mortality burdens, such as those from COPD, self-inflicted injuries and road traffic accidents. These can invoke the interest of non-health sector policy makers to get involved.

The strength of this study hinges on the complete vital registration systems, since the study regions are surveillance points for vital statistics in China. The problem of underreporting of deaths found in many studies is thus minimized. Underreporting of deaths has been shown to be more common in infant deaths in a previous study, especially in rural regions [38]. Had this been the case in the current study, the mortality disparity in group I causes would have been underestimated. This study is confined to mortality burden. While this type of burden declines with improvements to the social-economic status of a region, disability burden may be a more important problem in other contexts [39]. Such burden will be the focus of future research. Problems identified solely on the basis of mortality data may be underestimated. For example, in a study in Pakistan [16], injuries ranked eleventh according to YLL but second according to YLD, resulting in their ranking fifth based on DALY.

## Conclusion

The findings suggest that a strong health advocacy should be applied to rural regions of Yunnan province, especially on group I diseases and group III injuries. A continual and consistent effort in prevention and measures to reduce the burden from metabolic syndrome in all regions should be strengthened.

## Authors' contributions

Cai Le carried out the study and drafted the manuscript. Virasakdi Chongsuvivatwong conceptualized the research idea, participated in the design of the study, interpreted the results and helped to draft the manuscript. Both authors read and approved the final manuscript.
